# Strategies to improve outcomes of youth experiencing healthcare transition from pediatric to adult HIV care in a large U.S. city

**DOI:** 10.1186/s13690-023-01057-8

**Published:** 2023-03-31

**Authors:** Florence Momplaisir, Kassandra McGlonn, Megan Grabill, Kaelo Moahi, Hervette Nkwihoreze, Kayla Knowles, Roberta Laguerre, Nadia Dowshen, Sophia A. Hussen, Amanda E. Tanner, Elizabeth D. Lowenthal

**Affiliations:** 1 Department of Medicine, Division of Infectious Diseases, Perelman School of Medicine, University of Pennsylvania, 1201 Blockley Hall, 423 Guardian Drive, Philadelphia, PA 19102 USA; 2 Leonard Davis Institute of Health Economics, University of Pennsylvania, Philadelphia, PA USA; 3 Institute of Public Health, Epidemiology & Biostatistics, Florida A&M University, Tallahassee, FL USA; 4 Department of Family Medicine and Community Health, University of Pennsylvania Perelman School of Medicine, Philadelphia, PA USA; 5 Department of Pediatrics, University of Pennsylvania Perelman School of Medicine, Philadelphia, PA USA; 6 Department of Pediatrics, St Christopher’s Hospital for Children, Philadelphia, PA USA; 7 Division of Adolescent Medicine, Children’s Hospital of Philadelphia, Philadelphia, PA USA; 8 Hubert Department of Global Health, Emory University Rollins School of Public Health, Atlanta, GA USA; 9 Department of Public Health Education, University of North Carolina Greensboro, Greensboro, NC USA; 10 Department of Biostatistics, Epidemiology and Informatics, University of Pennsylvania Perelman School of Medicine, Philadelphia, PA USA

**Keywords:** Youth with HIV, Healthcare transition, Viral suppression, Pediatric care

## Abstract

**Background:**

The healthcare transition (HCT) from pediatric to adult HIV care can be disruptive to HIV care engagement and viral suppression for youth living with HIV (YLH).

**Methods:**

We performed qualitative interviews with 20 YLH who experienced HCT and with 20 multidisciplinary pediatric and adult HIV clinicians to assess and rank barriers and facilitators to HCT and obtain their perspectives on strategies to improve the HCT process. We used the Exploration Preparation Implementation Sustainment Framework to guide this qualitative inquiry.

**Results:**

The most impactful barriers identified by YLH and clinicians focused on issues affecting the patient-clinician relationship, including building trust, and accessibility of clinicians. Both groups reported that having to leave the pediatric team was a significant barrier (ranked #1 for clinicians and #2 for YLH). The most impactful facilitator included having a social worker or case manager to navigate the HCT (listed #1 by clinicians and #2 by YLH); case managers were also identified as the individual most suited to support HCT. While YLH reported difficulty building trust with their new clinician as their #1 barrier, they also ranked the trust they ultimately built with a new clinician as their #1 facilitator. Factors reported to bridge pediatric and adult care included providing a warm handoff, medical record transfer, developing relationships between pediatric clinics and a network of youth-friendly adult clinics, and having the pediatric case manager attend the first adult appointment. Longer new patient visits, increased health communication between YLH and clinicians and sharing vetted clinician profiles with YLH were identified as innovative strategies.

**Conclusion:**

In this multi-disciplinary contextual inquiry, we have identified several determinants that may be targeted to improve HCT for YLH.

**Supplementary Information:**

The online version contains supplementary material available at 10.1186/s13690-023-01057-8.

## Background

Youth (age 13–24) and young adults (25–29) share a disproportionate burden of HIV infection in the United States (U.S.) and experience poor HIV care continuum outcomes [1]. Although viral suppression has improved across most demographic groups in past decades, youth and young adults consistently continue to experience lower rates of viral suppression compared to older adults [2]. Healthcare transition (HCT) from pediatric to adult HIV care can be particularly disruptive to care engagement and viral suppression. Ideally, the preparation for HCT is a multi-disciplinary process that starts many years before the transfer out of pediatric care and includes an action plan tailored to the skills, social context, and needs of youth living with HIV (YLH) undergoing HCT. [3, 4] However, it’s unclear that this preparatory process is consistently applied and metrics for successful HCT are generally not captured or shared between pediatric and adult care clinics.

Barriers at multiple levels (individual, health system, and societal) contribute to poor health outcomes for YLH undergoing HCT. [5, 6] Existing HIV treatment guidelines provide a set of strategies to support HCT. Some include providing patient education in preparation for HCT, assigning a “transition point person” who can help the YLH navigate HCT, and providing a warm hand off [7]. Although such strategies can be helpful, we have a limited understanding of how well they are integrated in existing clinic workflows. We also have a poor understanding of the broad set of strategies, whether existing or new, that YLH and clinicians find helpful. This is particularly important as there are limited evidence-based practices aimed at supporting HCT of YLH. Here, we engage in a contextual inquiry of barriers and facilitators of HCT using YLH and clinician perspectives and inquire about strategies to improve HCT with the goal of improving HIV care continuum outcomes of YLH.

## Methods

### Implementation framework

We used the Exploration Preparation Implementation Sustainment (EPIS) Framework to guide our research [8]. The EPIS framework highlights the essential aspects of implementation that guide program development, identification, preparation, implementation and sustainment and incorporates bridging and innovation factors within and across levels of outer context (external to the HIV clinic) and inner context (specific to the HIV clinic) to inform the implementation process. In the context of this research, bridging factors focus on strategies that currently exist but need to be strengthened or used consistently during HCT while innovation factors focus on strategies that do not currently exist but emerged during the interviews as novel strategies to faciliate HCT.

### Study setting

This qualitative study was completed with clinicians across 3 pediatric clinics caring for the majority of YLH in the city of Philadelphia and 3 adult HIV care clinics where a significant proportion of YLH transition to adult care [9]. The population of YLH differs across the pediatric clinics with one clinic caring mostly for youth who acquire HIV during adolescence, another caring mostly for youth who acquire HIV perinatally, and the third caring for a mix of the two populations. All the pediatric clinics employ a preparatory process for HCT which involves assessing YLH’ readiness for HCT, addressing barriers and helping YLH build up the skills needed to succeed in adult HIV care. The pediatric care team identifies and recommends an adult clinic for care continuity using an individualized approach and by considering psychosocial factors (such as autonomy and self-efficacy) and structural factors (such as insurance and distance to new clinic). One of the pediatric clinics has an integrated pediatric-adult HIV care model where post-HCT, YLH attend the same clinic, retain their extended care team members (case managers, social workers, behavioral health consultants, etc.) and only the physician or advanced practice provider changes from a pediatric to an adult practitioner. For the purpose of this study, we use the word “clinician” to describe multi-disciplinary team members (physicians, nurses, case managers, social workers, youth counselor and behavioral health consultants).

### Recruitment and study procedures

The pediatric clinical teams routinely generate a list of patients who experienced HCT. This is done as part of the clinics’ quality improvement process and for reporting to the Philadelphia Department of Public Health. Clinical team members contacted patients who experienced HCT within the past 18 months and asked them for their interest in participating in the study. Among those who expressed interest, clinical team members obtained verbal permission to share their contact information (email and phone number) with our study team. YLH were eligible to complete the qualitative interview if they were 18 years of age or older at the time of the interview, had a diagnosis of HIV, established care at one of the three pediatric clinics at least 2 years before transitioning to adult care to ensure that they had been exposed to the HCT process. We captured YLH up to 18 months post-HCT to give them enough time to establish care in the adult clinic. We used purposive sampling [10] to recruit clinicians and clinic administrators across pediatric and adult clinics to ensure diversity of perspectives on barriers and facilitators to HCT.

Qualitative instrument for YLH. We asked open-ended questions on contextual determinants of the inner and outer setting that served as barriers and facilitators to a successful HCT and then asked each participant to rank barriers and facilitators, from most to least significant. We also asked YLH about their perspectives on strategies to improve the HCT, and to identify individuals within the clinical team or their social network, who would be best positioned to support their HCT. Following the interview, YLH completed a brief demographic questionnaire. They received $30 for completing the interview.

Qualitative instrument for clinicians. We asked clinicians to describe their role in the HCT, and provide their perspectives on inner and outer context barriers and facilitators to a successful HCT, including existing clinic resources and processes related to the HCT. Similar to the YLH instrument, we asked about their perspectives on strategies to improve the HCT and on individuals who can support this process. Clinicians also completed a basic demographic questionnaire. They were not compensated for their time.

The City of Philadelphia Institutional Review Board approved the study, and verbal informed consent was obtained from all study participants.

### Analysis

We followed the COnsolidated criteria for REporting Qualitative research (COREQ) for this work (Appendix) [11]. Interviews were conducted by members of the University of Pennsylvania’s Mixed Methods Research Lab (MMRL) [12]. The MMRL includes experts in qualitative and mixed-methods research. The interviews lasted between 20 and 45 min, took place over phone or video call, were conducted once, and only included the participant and the interviewer. The transcripts were not provided to participants for review. The interviewer took notes during the interviews that helped guide their reflective thinking. Participant identifying information was removed from the transcripts and the transcripts were entered into a customized qualitative NVIVO software database [13]. The NVivo database was used to store data, develop comprehensive coding schemas, code content, track emerging themes, and generate result summaries. We used a modified grounded theory approach for the data analysis [14]. We used the EPIS framework to select domains a priori and organized emerging themes into the EPIS framework. For the inductive analysis of the content, we used the constant comparative method [15] to sort and organize excerpts of raw data into groups according to attributes, and organize those groups in a structured way to identify emerging patterns and guide the identification of themes. The list of barriers, facilitators and the individual best suited to support the HCT were elicited from YLH and clinicians with open-ended questions and ranked based on their frequency.

## Results

We conducted 40 semi-structured interviews between January 24, 2020 and October 28, 2022: 20 interviews with YLH who experienced HCT and 20 interviews with multi-disciplinary clinicians from the pediatric and adult clinics. We reached thematic saturation between 12 and 15 interviews. We also interviewed one policy maker from the Philadelphia Department of Public Health’s (PDPH) Division of HIV Health (DHH) to obtain the perspective of End the HIV Epidemic (EHE) policymakers, acknowledging that we did not reach saturation with policy makers. Demographic characteristics of YLH and clinicians are included in Tables 1 and 2 respectively.

**Table 1:** Demographic Characteristics of Youth Living with HIV, Philadelphia, PA (January 2020- October 2022)

**Age (Years)**			
18–24	2 (12%)		
25–30	14 (88%)		
**Sex at birth**			
Male	7 (44%)		
Female	9 (56%)		
**Race**			
White	1 (6%)		
Black	15 (94%)		
**Ethnicity**			
Hispanic	1 (6%)		
**Education Status**			
GED/High school	3 (19%)		
Some College	9 (56%)		
Bachelor’s Degree	4 (25%)		
Adult clinicians Visit within 3 months of HCT*			
No	2 (12%)		
Yes	14 (88%)		
**Virally Suppressed post-HCT**			
Yes	11 (69%)		
No	4 (25%)		
Unknown	1 (6%)		

Out of 20 youth living with HIV, 4 participants did not fill the Redcap questionnaire.

*HCT: Healthcare transition.

**Table 2:** Demographic Characteristics of Pediatric and Adult HIV Clinicians, Philadelphia, PA (January 2020- October 2022)

Variables	n (%)
**Age**	
25–39	7 (35%)
40–54	10 (50%)
55+	3 (15%)
**Sex at birth**	
Male	3 (15%)
Female	16 (80%)
Missing	1 (5%)
**Race**	
White	12 (60%)
Black	7 (35%)
Asian	1 (5%)
**Ethnicity**	
Non-Hispanic	20 (100)
**Years of experience**	
0–10	7 (35%)
11–20	9 (45%)
21–30	4 (20%)

Clinicians included physicians (n = 6), nurses (n = 2), medical case managers (n = 3), social workers (n = 3), behavioral health consultant/youth counselor (n = 2), clinic administrators (n = 3) and a policy maker from the Philadelphia Department of Public Health (n = 1).

No clinicians identified as Hispanic/Latino, Native American/American Indian, Native Hawaiian/Pacific Islander, mixed race, or other.

**Fig. 1:**
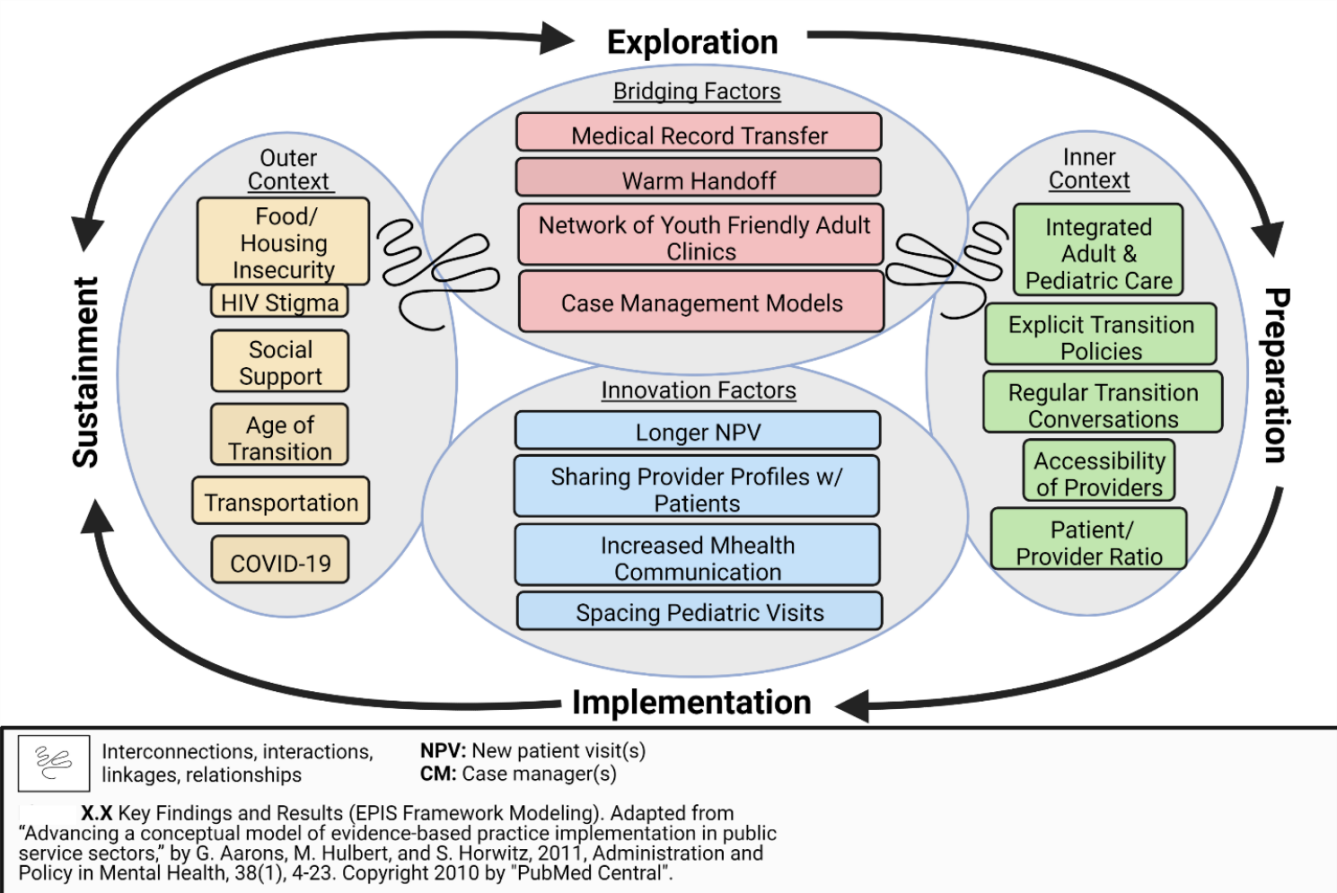
Summary of themes related to the Healthcare Transition (HCT) for youth with HIV, organized within the Exploration Preparation Implementation Sustainment (EPIS) Framework Dr. Aarons approved the use and adaptation of the EPIS figure for this work. Source: https://pubmed.ncbi.nlm.nih.gov/21197565/.

Below, we organized the themes that emerged across the domains of the EPIS framework and compared and contrasted YLH and clinician perspectives. Results are also summarized in Fig. 1.

### Inner context

#### Characteristics of pediatric care clinics

Clinicians and YLH described pediatric clinics as well-resourced, with small patient to clinician ratios and large interdisciplinary teams. These characteristics helped patients develop close-knit and long-standing relationships with clinicians. These relationships included many members of the clinical care team, such as physicians, case managers, nurses, and behavioral health staff. Pediatric clinics also had a large enough staff to check in on YLH regularly, making sure that they are aware of appointment times, assisting them with transportation to and from the clinic, and helping them with any insurance concerns that arise.

YLH 38: *(…) my pediatric doctor was still helping me with the problems that I had like filling my prescriptions, calling the insurance company, things like that. So, I didn’t, I didn’t feel it was urgent for me to move on to the adult doctor when I would gain all the assistance from this doctor.*

#### Characteristics of adult care clinics

Although adult clinics also used multi-disciplinary teams, resources that were commonly available to YLH in pediatric clinics were less available or were more challenging to access. On the other hand, adult clinicians also encouraged patient to have more autonomy with care engagement.

Adult clinician 1: *So, in some cases, transitioning to adult care actually helped their health because they felt more responsible for their care so they didn’t rely on anyone to check in on them to make sure they took their medicine for the week. It was, I know I have to do this so let me do it.*

#### Clinic policies, procedures, and resources impacting the HCT

Other inner context factors influencing HCT included clinic structure, resources, procedures, and transition practices. All pediatric clinics reported having explicit transition practices; however, the implementation and timing of implementation of these practices differed across clinics. For example, in the setting where integration of pediatric and adult HIV care occurred in the same clinic, HCT was easier.

YLH 27: *I want to say the transitioning part, switching over, that was really easy too […]. Not having to go through a hassle, a big obstacle like I had to go through a different hospital at all. Being able to stay at the same hospital, just a different doctor. That’s all.*

Clinics with non-integrated care used specific strategies such as extending the time that patient received support from a pediatric case management beyond HCT and having components of a formal transition checklist employed by multidisciplinary pediatric team members. Pediatric clinic resources and procedures such as frequent reminder calls, and community outreach, were noted to have a positive impact on HCT but their use varied across clinics.

Pediatric clinician 5: *We’ve also noticed that with the transition — and this is something that social work noticed a lot, too, was kind of following patients’ post-transition maybe for a short – like a shortened amount of time. It might be two months post-transition, before we can actually say that they have fully transitioned, right?*

The age requirement for HCT to occur at or before the age of 25 seemed to have a negative impact on YLH who were not prepared to make the leap to adult care. Adult clinics were not equipped to provide the same amount of support to YLH, both in terms of logistical support (i.e., case management, reminders, scheduling flexibility) and because they lacked the existing relationship with the patient.

YLH 36: *I think I’d been in pediatric care for like, an extra year, just because I just didn’t know how to let them go, and then I will admit, I kept on missing my appointment that I was supposed to have in order to get–my transitional appointment, I kept on missing it over and over and over and over and over. I never transitioned until maybe about 11 or 12 months after it was supposed to be over.*

The environment and policies of the adult clinics sometimes raised challenges for the YLH. One important policy consideration relates to health insurance coverage. While many pediatric clinics did not require that YLH use their own health insurance, adult clinics did. This was extremely challenging for YLH under the age of 26 who were still on their parent’s insurance plans and had not disclosed their HIV status to their family.

Pediatric clinician 11: *Private insurance through parents is a big barrier in transitioning care… Adult providers will not – if they have insurance, they have to use it and there is no flexibility with that either. And so that prevents youth who transition from here at 24, and are on their parents’ insurance until 26, that prevents them from getting care for almost two years and it is a big problem that we have faced.*

In addition, YLH described having fewer appointments in adult care settings with more time between visits. Some felt that the built-environment, particularly in the waiting room, needed to feel more welcoming.

### Outer context

The most prominent outer context factors were due to social determinants of health, HIV stigma, food insecurity, housing instability, and lack of social support. In addition, many YLH had not disclosed their HIV status to friends and family members and reported that it would be more challenging to do so with a new healthcare team.

Clinician 13: *Some of these young kids are dealing with being kicked out of homes. There’s homelessness. There may be substance use issues and so survival sex, and those kinds of things. I think other priorities, where I’m going to get my next meal, is going to be a higher priority than going to my HIV care.*

In addition, YLH experienced HCT at different stages of the COVID-19 pandemic and generally, were not able to evaluate the impact of the pandemic on HCT and did not report a significant delay in linkage to adult care due to the pandemic. Some YLH had their first adult HIV care visit via telemedicine and described it as “convenient”, while others found it to be rushed and unsatisfactory. Others had not used telemedicine and had all their visits in-person.

### Barriers to HCT

The most significant barriers affecting HCT that YLH and clinicians reported are summarized in Table 3 and occurred across the inner and outer setting. The most impactful barriers identified by YLH and clinicians focused on issues affecting the patient-clinician relationship, including building trust, and accessibility of clinicians. Both groups thought that having to leave the pediatric team was a significant barrier (ranked #1 for clinicians and #2 for YLH) due to the long-standing relationships established in pediatric care. Although clinicians brought up difficulty trusting a new clinician less frequently, this was the top-rated barrier for YLH.

YLH 29: *I stayed at [Children’s Hospital] as long as I possibly could because I didn’t want to go anywhere else.*

**Table 3:** Barriers to Healthcare Transition using Youth with HIV and Clinicians’ Perspectives

PATIENT BARRIERS				
1	**Difficulty Trusting New Clinician**			YLH might find it difficult to connect with a new clinician, particularly in comparison to their pediatric doctor. YLH also reported feeling judged for their medical history or reluctant to share sensitive and traumatic parts of their life.
2	**Leaving Pediatric Team**			Relationships with the patient’s pediatric team, particularly with doctors and case workers, are often emotionally significant. YLH can be reluctant to lose both the relationships and the additional logistical support.
3	**Communication Issues**			Adult clinicians have heavier caseloads and see YLH less often. YLH reported frustration both with slow response times and decentralized communication, both of which hinder attempts to build trust.
4	**Adjustment to Adult Healthcare System**			Particular elements of adult care, such as fewer appointments, moving to a new clinic, increased responsibility, or a general change in routine made it difficult for some YLH to transition.
5	**Appointment Scheduling**			Some YLH were frustrated with the wait time to see a doctor, others found it confusing to schedule appointments, and others were frustrated when the limited appointment times did not fit with their work schedule.
6	**Transportation**			Some YLH struggled to get to appointments on time or find parking. Others moved further away from their doctor or had difficulty traveling to their clinic.
	**CLINICIAN BARRIERS**			
1	**Leaving Pediatric Team**			Relationships between YLH and their pediatric clinicians are typically long-standing and emotionally significant. Transition is often hindered by general reluctance of both YLH and clinicians to break this bond.
2	**Accessibility of Clinicians**			Adult clinicians were described as being more difficult to engage with, noting that it may take weeks to get an appointment or require multiple steps to get a human on the phone when a patient requires assistance.
3	**Telling their Story**			YLH were often described by clinicians as being reluctant to “tell their story” to a new clinician. This reluctance is often discouraging enough to interfere with moving on to adult care.
4	**Forced Increased Autonomy**			Adult care comes with more autonomy for YLH and fewer support resources. YLH are not always prepared to take on these new responsibilities and they can fall out of care as a result.
5	**Difficulty Trusting New Clinician**			Connecting with an adult clinician is difficult for YLH as they grieve the loss of their relationship with their childhood clinician and adjust to a system that requires less face time than they are accustomed to.
6	**Developmental Stage**			YLH who are at transition age have variable levels of maturity and some are simply not ready to take on the new responsibilities of adult care, leading to an unsuccessful transition.

Youth living with HIV (YLH) and clinicians were asked to rank barriers to healthcare transition (HCT) from most to least significant. Answers were recorded and ordered according to how often they were brought up. The qualitative interviews took place across adult and pediatric care clinics in Philadelphia between January 2020 and October 2022.

For other barriers, clinicians tended to identify patient-level factors, such as YLH having to recount their stories, YLH’s autonomy, and their developmental stage; while YLH tended to report system-level factors, such as communication issues, appointment scheduling, adjustment to a new healthcare system, and issues related with transportation.

### Facilitators to HCT

The most significant facilitators affecting HCT are summarized in Table 4. YLH and clinicians identified having a social worker or case manager to help navigate HCT as a major facilitator (listed #1 by clinicians and #2 by YLH). These individuals were often described as trusted members of the care team who provided emotional and instrumental support to YLH.

YLH 35: *So, my social worker for my pediatric care, [social worker name 1], she played a big role in helping me with everything. So, without her, I don’t think I would even be this far in life. I honestly wouldn’t think so, because she was there and she made it so easy for me, because I didn’t know a lot about any of this. And she was the one person I would always go to and she would have the answers.*

**Table 4:** Facilitators to Healthcare Transition using Youth and Clinicians’ Perspectives

	PATIENT FACILITATORS		
1	**Trust in Adult Clinician**		YLH frequently mentioned that it was easier to transition and adjust to adult care when they were able to trust their adult clinician. YLH valued doctors who they felt made an effort to build a relationship with them and trusted their judgement when it came to care.
2	**Social Worker/Case Manager**		Case management services before, during, and after the transition helped YLH with logistical and emotional support. YLH often spoke highly of their relationship with their social worker.
3	**Warm Handoff**		Warm handoffs help establish a relationship with the new clinical team before leaving pediatric care while also leveraging the existing trust between the patient and their old team.
4	**Referral from Pediatric Clinicians**		Even in the absence of a warm handoff, YLH valued guidance on where to seek adult care and trusted that their pediatric doctor would direct them to a capable clinician.
5	**Maintaining Relationship with Pediatric Clinicians**		The relationship between patient and pediatric care team is frequently long-standing and emotionally significant. YLH mentioned that it was helpful to retain a relationship with their pediatric team even after transition.
6	**Same Location**		YLH who were able to stay in the same location appreciated the consistency in routine and the familiar surroundings.
	**CLINICIAN FACILITATORS**		
1	**Social Worker/Case Management**		Having access to case management services before, during and after the transition process creates vital support for YLH in moving to adult care successfully.
2	**Transition Preparation**		Being educated about the changes that come with transition before moving to adult care helps YLH to anticipate and successfully navigate them as they come.
3	**Similar Environment**		Transitioning within a clinic that has both pediatric and adult physicians eases the process for YLH as the physical environment and support staff remain the same.
4	**Warm Handoff**		Having a warm handoff between pediatric and adult clinicians assures that the patient has met and is comfortable with their new clinician and allows medical records to be transitioned between clinics.
5	**Adjusted to Diagnosis**		When YLH understand the responsibilities that come with their HIV diagnosis and are adjusted to what they need to do to manage their own health, transition will be more successful.
6	**Health Literacy**		The more YLH are taught about their health, resources available to them, and how to navigate the healthcare system, the more likely they are to transition successfully.

Youth living with HIV (YLH) and clinicians were asked to rank facilitators to healthcare transition (HCT) from most to least significant. Answers were recorded and ordered according to how often they were brought up. The qualitative interviews took place across adult and pediatric care clinics in Philadelphia between January 2020 and October 2022.

YLH and clinicians also ranked having a “warm hand-off”, where adult clinicians have the opportunity to meet the YLH prior to HCT and where communication between the pediatric and adult team is established prior to HCT, as a strong facilitator (#4 for clinicians and #3 for YLH).

YLH 25: *Some of the things that was easier was being able to meet with my doctor, my new primary care doctors, by setting up meetings through [pediatric clinic name]. Also meeting my new social worker and being able to feel comfortable before I start working with her. So, I would just say we just had like a great communication, I would say great communication between both parties, which I guess was the best thing ever.*

As seen with barriers, clinicians tended to focus on patient-level facilitators, such as adjusting to an HIV diagnosis and health literacy, while YLH focused on trusting the recommendation of the pediatric team for the choice of the adult clinic and keeping contact with the pediatric team.

### Individual to support the HCT

YLH and clinicians also identified supportive individuals who could facilitate the HCT. Each role and associated count are listed in Table 5. Case manager and social workers were identified as the most suited to providing social and instrumental support during the HCT.

**Table 5:** Clinicians’ and youth living with HIV’ perspectives on individuals best suited to support the healthcare transition

Clinicians’ Perspectives		YLH’ Perspectives	
Role	Count	Role	Count
Case Manager/Social Worker	18	Case Manager/Social Worker	18
Dependent on Patient Preference	6	Clinicians/prescribers	11
Entire Healthcare Team	4	Entire Healthcare Team	9
Family Member/Friend	2	Family Member/Friend	7
Clinicians/prescribers	2	Behavioral Health Staff	1
Behavioral Health Staff	2		

Prescribers are clinicians who prescribe antiretrovirals (physicians, nurse practitioners). The qualitative interviews took place across adult and pediatric care clinics in Philadelphia between January 2020 and October 2022.

### Bridging factors

Bridging factors included strategies used by pediatric and adult treatment teams; however, according to our findings, there was variability with implementation of these strategies, sometimes even within the same clinical program. Examples included providing a warm handoff, medical record transfer, developing relationships between pediatric clinics and a network of youth friendly adult clinics, and having the pediatric case manager attend the first adult appointment.

YLH who experienced a warm hand-off and were connected to adult clinicians before HCT praised the process, saying it built trust and increased comfort through the HCT. This took several forms; sometimes YLH met their adult care team in the pediatric clinic, through informal meetings, or were given information about a specific doctor. Some clinics encouraged the pediatric case manager to attend the first adult visit but this did not occur consistently.

YLH 21: *Yeah, because when I went over to my adult care, she had everything, like my doctor sent her over everything, I didn’t even have do much of anything because she sent that before my first appointment. So that made it easy, because she already had updated her on everything.*

It was important to many YLH that medical record sharing occurred so that adult clinicians would know their health history before the first adult visit. Networking between adult and pediatric clinics was noted as an important bridging factor as it helped foster a better fit by matching YLH to settings where their needs were most likely to be met. Some clinicians noted that certain adult clinicians had a special interest in YLH and paid particular attention to youth-specific needs and concerns.

### Innovation factors

Both YLH and clinicians provided examples of innovative strategies to support HCT, including having longer new patient visits, increased health communication, sharing vetted clinician profiles with YLH, and less frequent pediatric visits during the year prior to HCT for YLH who are virally suppressed.

According to some YLH, longer new patient visits were justified by being new to an adult clinic; they thought that this time was needed to be fully oriented into their new clinical setting. YLH and clinicians also suggested improving the accessibility of adult clinicians by expanding their means of communication to include text messaging. Though busy schedules are challenging to navigate in high-volume adult clinics, being able to contact clinicians quickly and efficiently through texting was noted as a significant way to improve communication between clinicians and YLH.

Clinician 15: *Texting is how people communicate in 2020, and so I think we need to acknowledge that and say like, ‘well we called you and left a message,’ or ‘the phone was not working,’ isn’t really fair.*

Sharing vetted profiles of adult clinicians eased the transition between pediatric and adult care and increased YLH’ access to information about the landscape of adult care before transition occurred. Information about insurance providers accepted in adult clinics was noted as particularly useful because it helped narrow down the landscape of potential clinics.

YLH 29: *Definitely having the [clinician] recommendation. They change and they was giving them my information, so they reached out to me. It wasn’t a search and find type of thing. Having the information was good. I moved back here, so I had to set up my healthcare and knew which one to pick because I knew which doctors, which insurance companies they took. Having the doctor’s information, where they were, and just knowing that they were a trusted source and it came from a trusted source made it all easy.*

Some clinicians thought that in cases where YLH were doing well, the common practice of “hand -holding” (e.g., allowing YLH to remain in pediatric care even if they are ready to transition) lengthened the transition process because the resources and close patient relationships that are common in pediatric clinics end up being a hindrance to patient independence. Knowing, for example, that clinicians will excuse being late to appointments and take care of insurance problems means that YLH had little incentive to manage logistics themselves. While handling insurance and appointment reminders for younger patients may make sense, adult clinicians explained that it begins to cause problems as patients get older. Thus, the suggestion to have less frequent pediatric appointments in the months or years leading up to the transition if YLH are doing well clinically (e.g., virally suppressed) to help them adapt to the expectations of adult care.

## Discussion

Given the importance of successful HCT in maintaining viral suppression in YLH, we conducted a study with emphasis on the pre-implementation phase of the EPIS framework to better understand multi-level factors that contribute to HCT challenges and successes. This study was based at pediatric and adult HIV clinics where most YLH in Philadelphia receive HIV care [9]. U.S. based studies show variability in successful linkage to adult care with linkage rates up to 12 months post-HCT varying between 37% and 84% based on the setting. [6, 16] Our contextual inquiry on inner and outer context determinants of HCT provides key findings to improve HCT of YLH going from pediatric to adult HIV care and adds to already documented barriers and facilitators to HCT. [6, 17, 18]

We found that the most impactful barrier to HCT centered around challenges building trusting relationships with the adult clinician and challenges leaving the pediatric team. This is reflected by the fact that leaving the pediatric team was ranked as the number 2 barrier to HCT by YLH, and the #1 barrier by clinicians. For YLH, difficulty establishing trust with the adult clinicians was the #1 barrier to HCT and this was compounded by issues related to communication and difficulty accessing clinicians. Establishing trusting patient-clinician relationships has been described as a strong facilitator for ART adherence and retention in care across the adolescent HIV literature, including during HCT, [19] and in the larger population of people with HIV. [20–22] Therefore, strategies are needed to improve the patient-clinician communication for YLH transitioning to adult HIV care. A scoping review of interventions aimed at providing ART adherence support for YLH show that most interventions include individual counselling, support groups, family-centered services, and treatment supporters (such as caregivers) but none focused on clinicians [23]. Although clinician-based interventions for this population have not been well established, our results suggest that strategies focused on improving clinician access and communication might help build trust. Another consideration is to create a mechanism for immediate patient feedback post-HCT measuring effective communication with adult clinicians and sharing the results with adult and pediatric teams to (1) allow the adult clinics to more readily address the YLH’s concerns and (2) to allow the pediatric clinics to modify their referral networks with adult clinics based on patient feedback. In addition, the contrast between individual-level barriers raised by clinicians and system-level barriers raised by YLH demonstrate the importance of gathering a diversity of perspectives in the pre-implementation phase of this work to ensure that proposed strategies comprehensively address existing barriers to successful HCT.

In addition, inner context factors contrasted across the pediatric and adult care settings reflecting key cultural and resource differences across these settings [24]. Resources such as case management, behavioral health services, and primary care were felt to be more easily integrated in pediatric HIV clinics. The physical environment was different as well: some YLH felt that adult clinic waiting rooms were not as welcoming. This finding is important as an analysis from HIV Research Network clinics demonstrated that YLH attending clinics that included youth-friendly structures of care were almost twice as likely to be retained in care compared to YLH who did not attend youth-friendly clinics [25]. Clinic-level factors that were most influential on care retention were having youth-friendly waiting area, evening clinic hours, and clinicians with adolescent health training. [17, 25].

Social determinants that YLH experienced made them even more vulnerable to experiencing poor HIV care continuum outcomes. Those experiencing food and housing insecurity seem to be particularly vulnerable during HCT given resources that aim to address these factors changed as YLH transitioned from pediatric to adult care. Our findings further support the role of effective case management during HCT and continued efforts to address housing instability and other social determinants negatively impacting care [26]. In addition, models of case management need to be tested for this population, including having the pediatric case manager continue to follow the YLH for a specified time post-HCT, or having adult clinics staffed with youth-specific case managers, compared to standard case management.

Several bridging factors were identified in the qualitative interviews, most related to practices that should be considered standard of care such as sharing of medical records and providing a warm handoff, as detailed in the Got Transition 6 Core Elements [27]. However, there was variability in implementation across clinics. This finding is supported by other studies showing that inconsistencies in implementation of HCT protocols. [6, 28] A prospective study of fourteen clinics from the Adolescent Trial Network evaluated the use of a “checklist” to support the HCT of YLH. All the items included in this checklist emerged as facilitators in our interviews. They include having a transition protocol, identifying a staff person to support the HCT (YLH and clinicians overwhelming identified a case manager or social worker for that role), providing a warm hand-off by having an adult clinician come to the pediatric clinic or a having a member of the pediatric clinical team attend the first adult appointment, providing information about adult clinic options, addressing health insurance issues, and the model of care delivery (i.e., integrated pediatric-adult care or not) [6]. Clinics who applied this checklist with consistency were 5 times more likely to have over 50% of their YLH successfully transition to adult care, defined as having at least one adult clinic appointment during the 9-month study period [6]. Finally, several innovative factors were identified; however, they will require adjustment in clinic work flow and need to be piloted for feasibility and acceptability.

Our study has several limitations. The perspectives shared by YLH and clinicians are not necessarily generalizable to other contexts which may differ in patient population, relationships between adult and pediatric centers, local resources available to YLH, and social determinants most significantly impacting YLH. While the study was able to enroll from 3 local pediatric HIV centers, it did not capture the perspective of clinicians from smaller clinics in Philadelphia. In addition, all study participants were linked to adult HIV care. Views of those who did not successfully experience HCT are not included in this study. Furthermore, we did not investigate the mechanisms of the negative impact of outer context factors (HIV stigma, food insecurity and housing instability, and lack of social support) on HCT. More work needs to be done to understand these mechanisms and test strategies to mitigate their impact on HCT.

## Conclusion

In conclusion, our work identified barriers and facilitators to HCT from the perspective of YLH and clinicians at pediatric and adult clinics that need to be taken under consideration to improve the transition process and maximize HIV treatment success for YLH entering adult HIV care. We also identified bridging and innovation factors that may require testing of case management models, improving communication between YLH and clinicians, and adjustment of workflows to better support youth undergoing HCT.

## Electronic supplementary material

Below is the link to the electronic supplementary material.

COREQ (COnsolidated criteria for REporting Qualitative research) Checklist

## Data Availability

available upon request.
